# Supplementary material for: Fabrication of Anatase TiO2/PVDF Composite Membrane for Oil-in-Water Emulsion Separation and Dye Photocatalytic Degradation
Source: Membranes (Basel). 2023 Mar 22;13(3):364. doi: 10.3390/membranes13030364 (PMC10055995; doi:10.3390/membranes13030364)
Supplement: Supplementary file 1 [file membranes-13-00364-s001.zip › membranes-2268658-supplementary.pdf]

membranes

Supplementary Materials

Fabrication of Anatase TiO_2/PVDF Composite Membrane for Oil-in-Water Emulsion Separation and Dye Photocatalytic Degradation

Chengcai Li [1], Hewei Yu [1], Biao Huang [1], Guojin Liu [1,2], Yuhai Guo [1,3,*], Hailin Zhu [1,2] and Bin Yu [1,*]

[1] Zhejiang Provincial Key Laboratory of Fiber Materials and Manufacturing Technology, Zhejiang Sci-Tech University, Hangzhou 310018, China
[2] Zhejiang Provincial Innovation Center of Advanced Textile Technology, Shaoxing 312000, China
[3] Zhejiang Sci-Tech University Huzhou Research Institute Co., LTD, Huzhou 313000, China
* Correspondence: gyh@zstu.edu.cn (Y.G.); yubin7712@163.com (B.Y.)

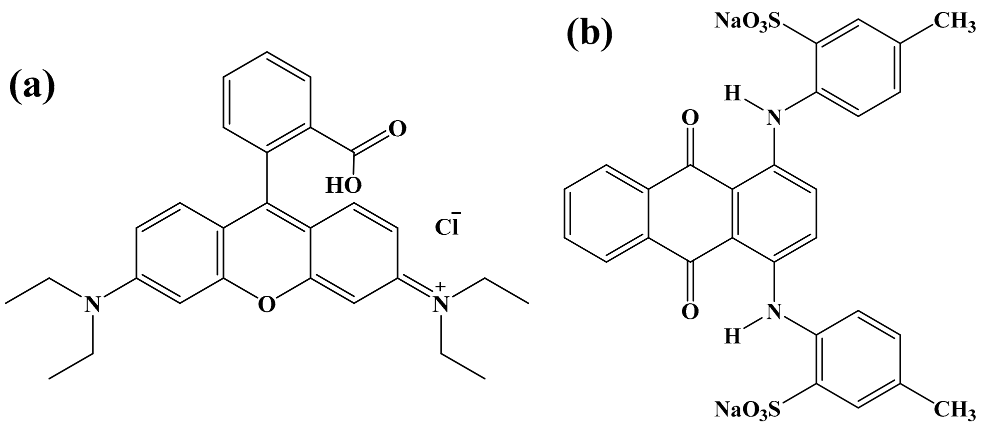

Figure S1. Chemical structure of RhB and AG-25 dyes.

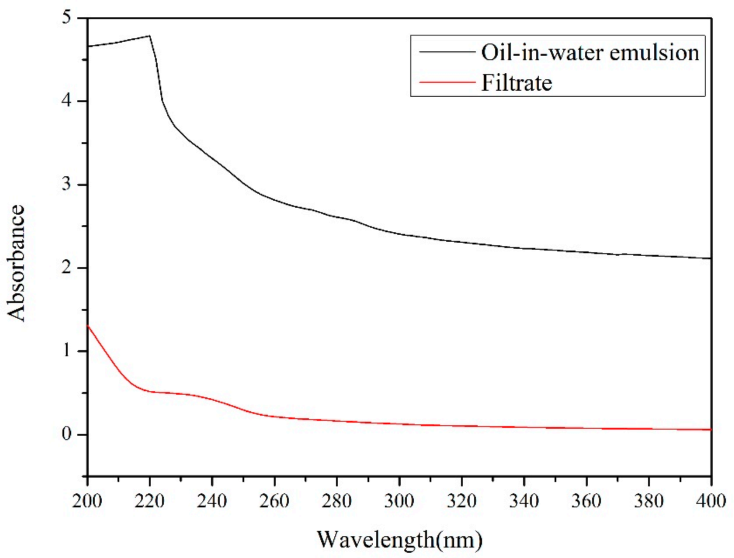

Figure S2. The maximum UV absorption wavelength of soybean oil.

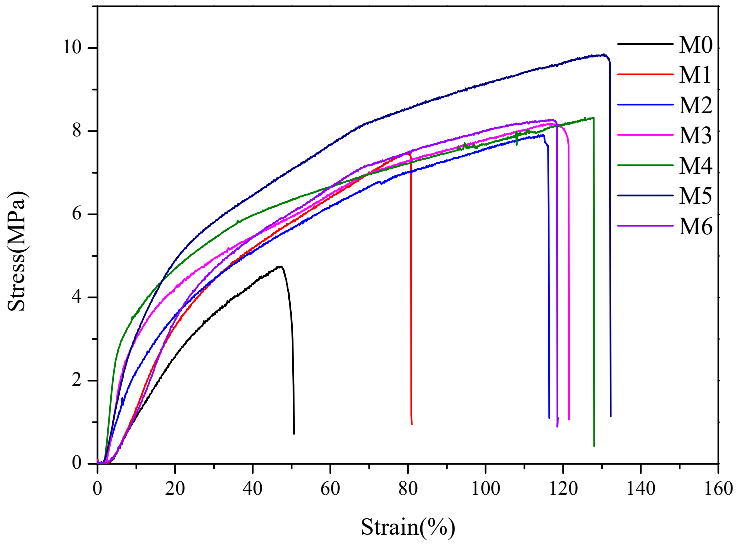

Figure S3. The membrane stress-strain curves of different PEMA content.

Obviously, the composite membranes show an increased tensile strength and tensile strain compared with pristine PVDF membrane, indicate the blending of PEMA can enhance the mechanical strength of membranes. Additionally, the tensile strength (from 4.75 Mpa to 9.84Mpa) and tensile strain (from 47.37% to 130%) increase gradually with the PEMA content increasing from 0 wt% to 25 wt %. Nevertheless, for the 30 wt% PEMA content, the tensile strength (form 9.84 Mpa to 8.28 Mpa) and tensile strain suddenly decreased (from 130% to 117.38%), indicating the excessive blending PEMA into PVDF membrane matrix will reduce the flexibility of the membrane. In conclusion, the composite membrane has excellent mechanical properties.

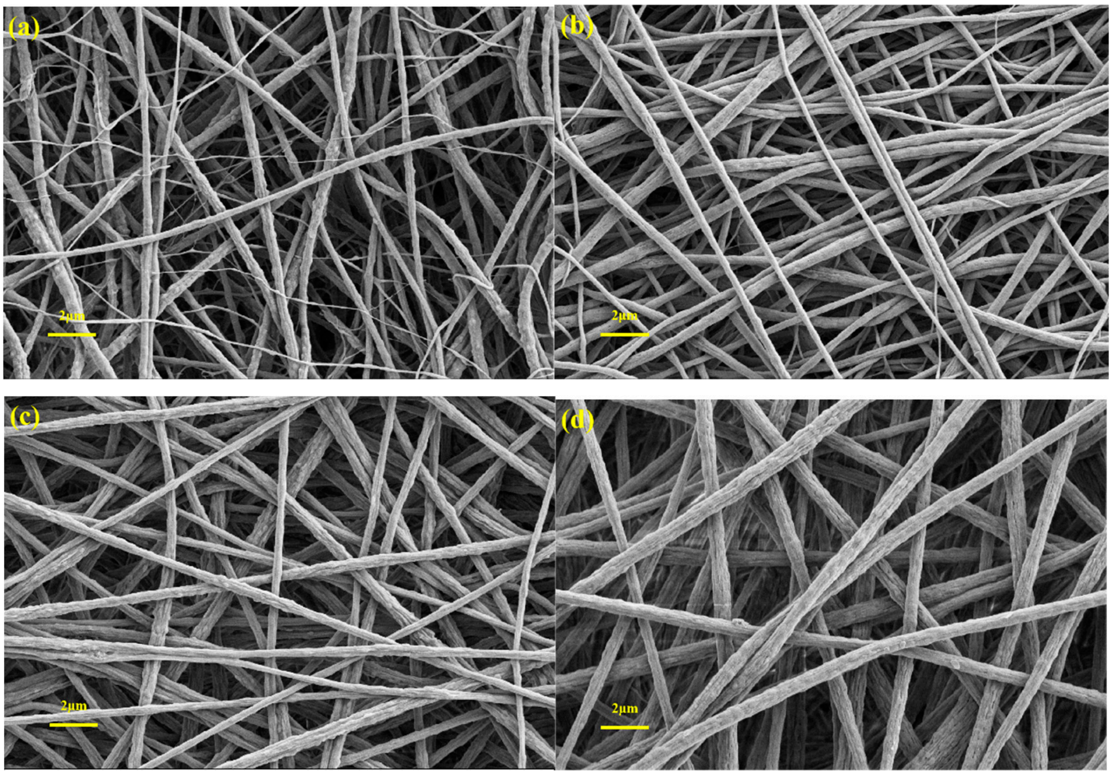

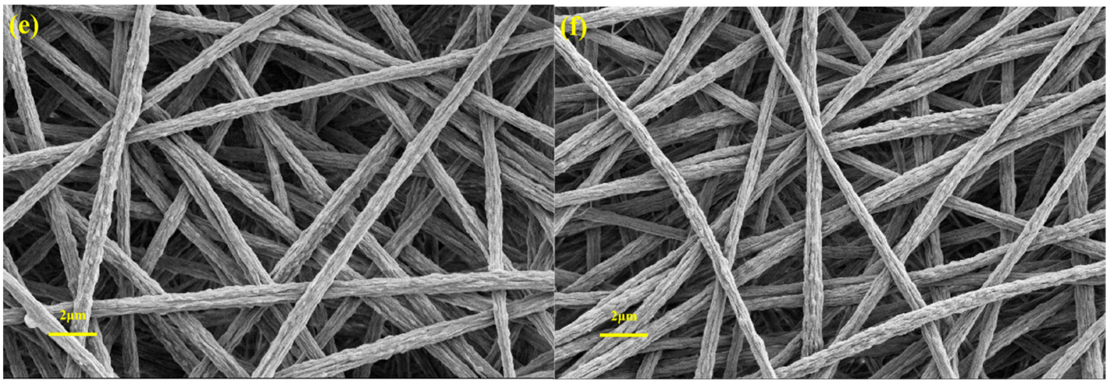

Figure S4. The membraen SEM of different PEMA content. (**a**) 5%, (**b**) 10%, (**c**) 15%, (**d**) 20%, (**e**) 25% and (**f**) 30%.

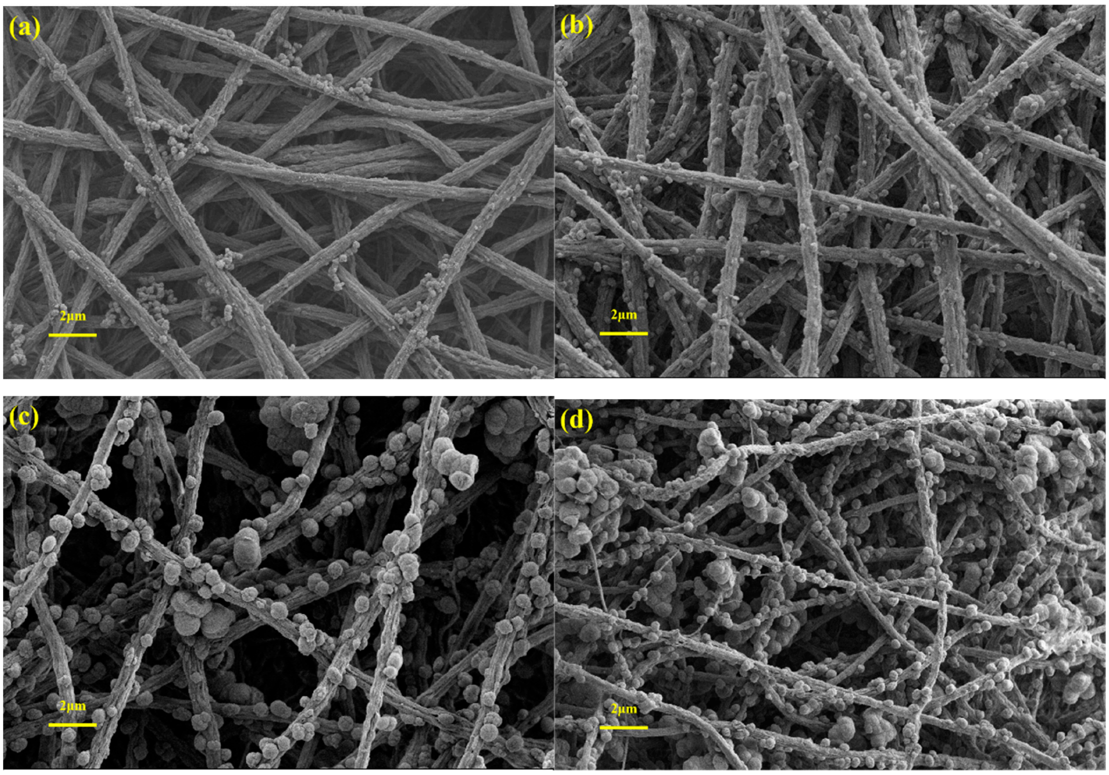

Figure S5. The membrane SEM of different TiO_2 deposition time. (**a**) 1h, (**b**) 2h, (**c**) 3h and (**d**) 4h.

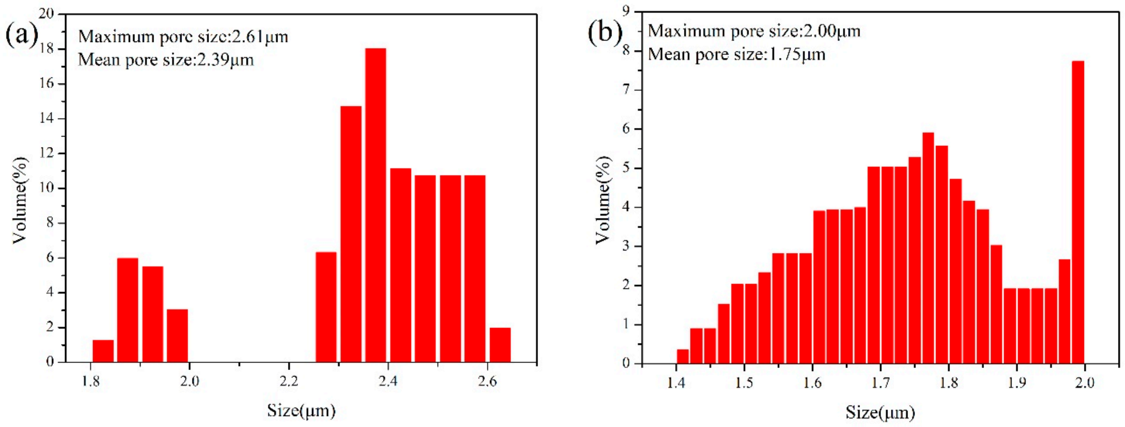

Figure S6. The pore size distribution map of M5(**a**) and M5-TiO_2(2) membrane.

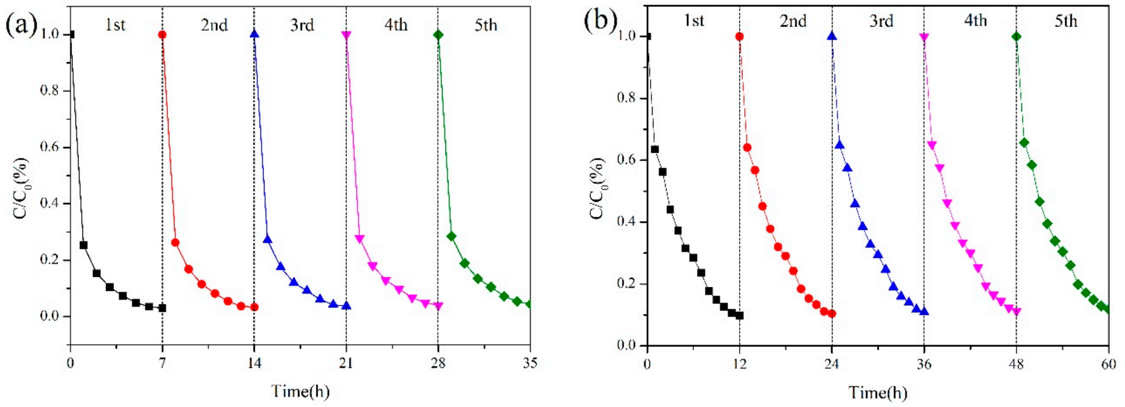

(**a**) (**b**)

Figure S7. Adsorption-degradation performance of the M5-TiO2(2) for RhB (**a**) and AG-25 (**b**) during 5 cycles.

Table S1. Comparison of the emulsion separation performance and dye photocatalytic degradation performance of this work with related literature.

Membrane	Emulsion	Separation Efficiency (%)	Flux·(L·m^{-2}·h^{-1}·bar)	Photocatalytic Efficiency (%)	Reference
M5-TiO2(2)	O/W 1:1000 (m/m)	98.95	6786.64	97%(RhB 5ppm) 90.2%(AG-25 50ppm)	This work
PTFE-PGAL/PEI-KH560(1.2%)	O/W 1:1000 (m/m)	98.2	3484.46	NA	[3]
PVDF/ZnO:La(37 %)	NA	NA	NA	93.36% (RhB 5ppm) 96.33 (MB 10ppm)	[9]
PVDF/NM88B-1	O/W 1:100 (V/V)	>99.4	340-1970	97% (MB 10ppm) added with H2O2	[17]
Janus F-TiO_2 @PPS membrane	O/W 1:100 (V/V)	>98	711-1055	NA	[42]
PP-Si-PAMAM/PVDF-2	O/W 1:100 (V/V)	99.1>	922-1634	88% (RhB 5ppm) 90% (MB 5ppm) added with H2O2	[43]
CuWO4/Cu2O coated mesh	O&W mixture 3:7 (V/V)	95	NA	23.9% (MB 0.1mM) 90.2% (MB 0.1mM) added with Na2S2O8	[44]

Reference

[3] Li, C.; He, Z.; Wang, F.; Zhu, H.; Guo, Y.; Chen, M. Laccase-catalyzed homo-polymer of GAL and cross-linking with PEI to enhance hydrophilicity and antifouling property of PTFE flat membrane. *Prog. Org. Coatings* **2019**, *132*, 429–439, https://doi.org/10.1016/j.porgcoat.2019.03.038.

[9] Pascariu, P.; Cojocaru, C.; Samoila, P.; Olaru, N.; Bele, A.; Airinei, A. Novel electrospun membranes based on PVDF fibers embedding lanthanide doped ZnO for adsorption and photocatalytic degradation of dye organic pollutants. *Mater. Res. Bull.* **2021**, *141*, 111376

[17] Xie, A.; Cui, J.; Yang, J.; Chen, Y.; Lang, J.; Li, C.; Yan, Y.; Dai, J. Photo-Fenton self-cleaning PVDF/NH2-MIL-88B(Fe) membranes towards highly-efficient oil/water emulsion separation. *J. Membr. Sci.* **2019**, *595*, 117499, https://doi.org/10.1016/j.memsci.2019.117499.

[42] Yang, C.; Han, N.; Han, C.Y.; Wang, M.D.; Zhang, W.X.; Wang, W.J.; Zhang, Z.X. Design of a janus F-TiO2@PPS porous membrane with asymmetric wettability for switchable oil/water separation. *ACS Appl. Mater. Interfaces* **2019**, *11*, 25, 22408–22418.

[43] Zhang, H.; Xu, M.; Chen, M. N.; Cui, Y.H.; Chen, L.; Dai, X.H.; Dai, J.D. Fabrication of high flux porphrin-cored with silox-ane-poly(amido amine) dendrimer/PVDF composite membrane for oil/water separation anddye degradation. *J. Environ. Chem. Eng* **2022**, *10*, 107634

[44] Zhou, C.; Cheng, J.; Hou, K.; Zhu, Z.; Zheng, Y. Preparation of CuWO4@Cu2O film on copper mesh by anodization for oil/water separation and aqueous pollutant degradation. *Chem. Eng. J.* **2017**, *307*, 803–811, https://doi.org/10.1016/j.cej.2016.08.119.
